# A Study of the Occurrence of Aflatoxin M1 in Milk Supply Chain over a Seven-Year Period (2014–2020): Human Exposure Assessment and Risk Characterization in the Population of Central Italy

**DOI:** 10.3390/foods10071529

**Published:** 2021-07-02

**Authors:** Rossana Roila, Raffaella Branciari, Emanuela Verdini, David Ranucci, Andrea Valiani, Alessandro Pelliccia, Laura Fioroni, Ivan Pecorelli

**Affiliations:** 1 Department of Veterinary Medicine, University of Perugia, Via San Costanzo 4, 06121 Perugia, Italy; rossana.roila@unipg.it (R.R.); david.ranucci@unipg.it (D.R.); 2 Istituto Zooprofilattico Sperimentale dell’Umbria e delle Marche “Togo Rosati”, 06126 Perugia, Italy; e.verdini@izsum.it (E.V.); a.valiani@izsum.it (A.V.); a.pelliccia@izsum.it (A.P.); l.fioroni@izsum.it (L.F.); i.pecorelli@izsum.it (I.P.)

**Keywords:** margin of exposure, cow milk, ewe milk, cheese, public health, dietary exposure, official control, risk assessment

## Abstract

Aflatoxin food contamination represents a rising global issue that will continue to increase due to climate change. Aflatoxin M1 (AFM1) is of high concern for the whole dairy industry. In light of AFM1′s harmful potential, a human health exposure assessment and risk characterization were performed for all age populations of central Italy with regard to milk and cheese consumption by means of the margin of exposure (MOE). In total, 16,934 cow and ewe’s milk samples were collected from 2014 to 2020 and analyzed by an enzyme-linked immunosorbent assay (ELISA) screening method, confirmed by high-performance liquid chromatography with a fluorescence detector (HPLC-FLD). The average concentration of AFM1 in cow’s milk ranged from 0.009 to 0.015 µg/kg, while in ewe’s milk, the average concentration ranged from 0.009 to 0.013 µg/kg. The average amount of AFM1 exposure ranged from 0.00005 to 0.00195 g/kg bw/day, with the main contributor represented by drinking milk, followed by the consumption of soft cheeses. A high level of public health concern related to the youngest consumers has arisen from risk characterizations highlighting the need for constant monitoring of AFM1′s occurrence in milk by inspection authorities, alongside regular updates with regard to exposure assessments.

## 1. Introduction

Mycotoxin contamination has been habitually considered as a problem that occurs in economically developing countries. However, climate change is anticipated to enhance the risk in industrialized countries, including Europe, by having a drastic impact on the presence of these toxins in food [1]. In particular, aflatoxins (AFs) represent a rising global health concern as an unavoidable and unpredictable problem, even in countries where good agricultural, storage, and processing practices are optimized and fully implemented. As recently reported by the European Food Safety Authority (EFSA), the occurrence of aflatoxins in foodstuffs should be constantly monitored [1,2,3,4,5]. Toxigenic strains of *Aspergillus* spp. fungi are mainly responsible for the production of AFs in many food ingredients and feed materials, such as nuts and grains [6]. In addition to their harmful potential in terms of public health, AFs pose a significant economic concern as a burden on agriculture and international trading [7,8]; AFs, for instance, are most commonly mycotoxins that are associated with RASFF (Rapid Alert System for Food and Feed) notifications and are the main hazard cited by the EU in foodstuff border rejections [9]. Aflatoxin B1 (AFB1) is the most frequent and is amongst the most potent genotoxic and carcinogenic fungal toxin found in feed and food. If ruminants are fed contaminated feed, the ingested AFB1 is partly degraded by the forestomach and partly metabolized by the liver into monohydroxy derivatives, mainly 4-hydroxylated aflatoxin M1 (AFM1), then excreted into the milk through the mammary glands [6,10]. The excretion rate of AFM1 into milk varies between 0.3 and 6% of ingested AFB1, depending on the species and variety of the animals in question and the amount of milk produced [11,12]. In general, high-yielding breeds have higher carryover rates [13]. Although less potent than AFB1, AFM1 presents similar toxicological hazards and, due to its harmful potential, the International Agency for Research on Cancer (IARC) has classified this toxin as a group 1 human carcinogen [14], while the European Union has set the maximum level of AFM1 in consumable milk as equal to 0.050 µg/kg and infant formulae as equal to 0.025 µg/kg [15] to reduce human exposure to the minimum and most reasonably achievable level. Besides the contamination of raw milk, AFM1 is a matter of concern for the whole dairy production chain. AFM1 contamination in dairy products results from indirect milk contamination, as AFM1 is found in dairy products at levels that are 3−8-fold higher than in milk [16]. Specific maximum levels for AFM1 in dairy products, such as cheese, are still lacking. Nevertheless, dairy products must be obtained using milk compliant with the above AFM1 limits [10]. Furthermore, according to Article 2 of EC Regulation No. 1881/2006, the concentration factor is an important parameter that has to be established to evaluate the maximum level of contaminants in dried, diluted, processed, and composed foodstuffs, aiming to ensure that cheese has been produced from compliant milk [15].

Furthermore, in compliance with the aforementioned EC Regulation 1881/2006, when the competent authority carries out an official, control a specific concentration or dilution factors for the processed foodstuffs shall be provided and justified by the food business operator (FBO) [15]. In the case that FBO does not provide a proper concentration factor, to assure public health protection, the Italian Ministry of Health established the concentration factors on the basis of different cheese categories defined by the EC Decision of 18 December 1996 [17].

Italians are high consumers of milk and cheese, and, among the entire Italian population, infants and children have a higher intake of dairy food compared to adults, and hence are more exposed to the toxic substances present in milk [18].

The prevention and management of AFM1 contamination of milk used in dairy products is a priority issue due to potential concerns with regard to consumers’ health. In this context, a thorough risk assessment is crucial to ensure the safety of milk and dairy products, as they have a direct impact on public health. The aim of this study was to assess the occurrence of AFM1 in cow’s milk destined for use as drinking milk and in ewe and cow’s milk destined for use in cheesemaking produced in central Italy over a seven-year period. The human exposure to AFM1 was determined and a related risk characterization was performed for all age groups of the population.

## 2. Materials and Methods

### 2.1. Sampling Plan

A total of 16,934 ewe and cow’s milk samples were collected during a seven-year period, ranging from 2014 to 2020, within the framework of the official control and self-control plan of the Italian dairy industry. Samples were collected from more than 95% of the milk-producing farms in the Umbria region (central Italy), ensuring a very high representativeness of the local production of milk and products thereof. Sampling was performed in accordance with point F of Commission Regulation (EC) No 401/2006 [19] and analyzed by the Istituto Zooprofilattico Sperimentale of Umbria and Marche “Togo Rosati”.

The milk collected from several ewe and cattle farms is used both for the production of milk and for cheesemaking, which is carried out in processing plants located in the Umbria region.

### 2.2. Analytical Determinations

The milk samples were analyzed by an enzyme-linked immunosorbent assay (ELISA) screening method for AFM1 (first level control by a screening test), and samples above 0.050 µg/kg (suspected non-compliant) were confirmed by high-performance liquid chromatography with a fluorescence detector (HPLC-FLD). Both analytical methods are accredited according to ISO/IEC 17025:2018 requirements [20,21].

Sample preparation was carried out according to the instructions of the manufacturer. An I’Screen AflaM1 kit from Eurofins Technologies (Budapest, Hungary) was used for the ELISA and the following are the characteristics declared by the producer: limit of quantification: (LOQ) 0.005 µg/kg; average recovery: 80–140%; specificity (cross-reactivity): AFM1 100%. An in-house validation experiment, considering the mean recovery, repeatability, and intra-laboratory reproducibility, was carried out to verify the analytical performance of the test declared by the producer. Moreover, to ensure the analytical performance of the ELISA kit over time, every year, the laboratory participates in a proficiency test organized by Test Veritas (Padova, Italy) to verify the analytical performance of the ELISA kit. Each year, two samples are analyzed, and all results thus far have been satisfactory (|z| ≤ 2.0). Analyses of samples were conducted in duplicate, and repeatability was monitored. In each analytical batch, one negative and a two positive control samples were analyzed to check for the absence of interference and recovery. A milk sample with AFLM1 below the LOQ (0.005 µg/kg) was used as the negative control and two reference materials, with a concentration around the LOQ and the maximum level fixed by the law (0.050 µg/kg), respectively, were used to check for recovery [15].

For confirmation purposes, the AFM1 concentration was determined by HPLC-FLD in suspected non-compliant samples according to Pecorelli et al. [16]. Briefly, milk samples were defatted by high-speed centrifugation (2700× *g*, at 4 °C for 40 min), and subsequently 50 g of sample was purified by immunoaffinity columns (IAC, Easi-Extract Aflatoxin RP71/RP70N from R-Biopharm, Darmstadt, Germany). Twenty milliliters of phosphate-buffered saline (PBS) was applied on the column to wash it. Elution of AFM1 was performed in two steps: 1.25 mL of ACN/MeOH at 6/4 (*v*/*v*) was applied to ensure antibody denaturation and aflatoxin release, followed by 1.25 mL of water to quantitatively collect the toxin. The eluate was evaporated to dryness using a gentle stream of nitrogen and resuspended in 0.25 mL of water. Ten microliters of purified extract are injected in the UPLC-FLD system. Chromatographic separation was performed using a Kinetex C18 analytical column (50 × 2 mm; particle size 5 μm) from Phenomenex (Torrance, CA, USA) with a ternary gradient [Water (A); ACN (B) and MeOH (C)] at a flow rate of 0.8 mL/min. Initial conditions (71% A, 7% B, and 22% C) were kept for 1.25 min, and then the percentage of A was linearly decreased to 0%, while B was increased to 20% and C to 80% in 0.02 min. These conditions were kept until 1.6 min, and then the composition was restored to the initial ratio in 0.05 min and kept for 3.35 min until the end of the run to ensure removing interfering substances from the column. FLD (RF-20A XS, Shimadzu, Kyoto, Japan) conditions were set at λex = 360 nm, λem = 440 nm. The performance of the analytical procedure, for the determination of AFM1 in milk, was established in terms of the LOD, LOQ, linearity, recovery, and precision in both repeatability and intra-laboratory reproducibility conditions.

The left-censored data (results < LOQ) were handled using the substitution method, as suggested in the literature for studies in the field of food safety [22,23], particularly in relation to dietary exposure assessments of chemical substances [24]. The document suggests that the lower bound (LB) and upper bound (UB) approach should be used for chemicals likely to be present in food, such as mycotoxins [24]. The LB is obtained by assigning a value of zero to all samples reported as <LOQ, while the UB is obtained by assigning the numerical value of the LOQ to values reported as <LOQ.

### 2.3. Dietary Exposure Assessment

To estimate AFM1 dietary intake, a deterministic approach was applied by combining the normalized daily intake of milk and cheese with the mean concentration of AFM1 in milk. Detailed food consumption data were extrapolated from the latest version of the Comprehensive Food Consumption Database of the EFSA [25] on the basis of the 50th and 99th percentiles of Italian surveys of each population group: toddlers (≥12 months to <36 months old, weighing 11 kg), children (≥36 months to <10 years old, weighing 26 kg), adolescents (≥10 years to <18 years old, weighing 53 kg), adults (≥18 years to <65 years old, weighing 70 kg), and the elderly (≥65 years, weighing 70 kg). In order to define the contribution of dairy products to AFM1 human dietary exposure, for milk destined for use in cheesemaking, we took into consideration specific concentration factors with respect to the use of milk in a specific cheese category [26]. The cheese category was established followed the EU Decision of 18 December 1996 [17], which categorized cheeses by their moisture content on a fat-free basis (MFFB), identifying five different cheese categories: soft (MFFB ≥ 68%), semi-soft (68% > MFFB ≥ 62%), semi-hard (62% > MFFB ≥ 55%), hard (55% > MFFB ≥ 47%), and very hard (MFFB < 47%). For the five cheese groups, the AFM1 concentration factor was defined by a note issued by the Italian Health Ministry (Italian Health Ministry, 2019): 6 for very hard, 5 for hard and semi-hard, 4 for semi-soft, and 3 for soft cow cheese [27]. For ewe cheese in accordance with EC Regulation 1881/2006 as reported also in the note of Italian Health Ministry (Italian Health Ministry, 2013), we applied a concentration factor of 4.1, provided and justified by the food business operator [28].

The values of chronic dietary exposure (DE) to AFM1 from the selected population consumption data were combined with the AFM1 occurrence data [29,30], and DE was calculated according to the formula reported in literature [31] and adjusted as follows:

$$DE_{i}=\overset{n}{\underset{k-1}{\sum }}\left(\frac{I_{ik\;}\;C_{k}}{BW_{i}}\right)$$

*DEi* is the total dietary exposure to AFM1 of subject *i* (mg/kg bw/day), *I_i__k_* is the intake of food item *k* by subject *i* (g/d), *C_k_* is the AFM1 concentration of food item *k* (mg/kg), *BW_i_* is the mean body weight of subject *i* (kg), and *n* is the total number of food items consumed by subject *i* among the foods analyzed. In order to pursue the principle of public health protection, we applied the worst-case scenario for dietary exposure assessment; therefore, only the UB values of the AFM1 concentrations in milk were considered.

### 2.4. Risk Characterization

In order to esteem the severity of the public health concern about exposure to AFM1 through the consumption of milk and dairy products, we performed a risk characterization by applying the Margin of Exposure (MOE) approach, as suggested in the literature for substances that are both genotoxic and carcinogenic [32], by applying the following formula:

$$MOE=\frac{BMDL_{10}}{DE}$$

The MOE approach does not represent a precise quantification of risk; however, it provides an indication of the level of health concern about a substance’s presence in food, representing a valuable tool for risk managers in prioritizing risk management decisions [32]. The EFSA identified the liver carcinogenicity of aflatoxins as the pivotal effect on the risk assessment; therefore, the benchmark dose lower confidence limit for a benchmark response of 10% (*BMDL*_10_) with regard to the incidence of hepatocellular carcinomas (HCCs) in male rats was considered [1]. In the absence of a specific *BMDL*_10_ for AFM1, the *BMDL*_10_ for HCCs related to the ingestion of AFB1 (0.4 µg/kg body weight per day) was used in the present study for the definition of MOE applying a potency factor of 0.1, as reported by the EFSA [1,32]. According to the EFSA, MOE values of 10,000 or higher are of low concern from a public health point of view [32].

### 2.5. Uncertainty Analysis

To provide a more appropriate estimate of exposure for the specific scenario of the present study, we performed a thorough evaluation of inherent uncertainties according to the literature [22,31,33]. The analysis was conducted with the tiered method by applying the first tier (qualitative analysis of uncertainties), according to which the uncertainties affecting the dietary exposure assessment were identified and characterized [33]. The direction (over- or underestimation) and magnitude (the extent of the contribution) of each individual uncertainty, as well as the combined effect of all the uncertainties, were considered and are reported in the Supplementary Materials.

## 3. Results

**Figure 1:**
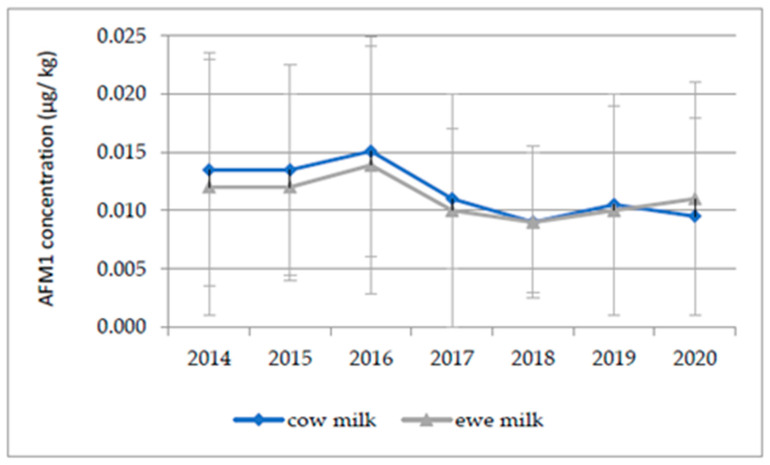
Yearly summary the mean AFM1 concentration and standard deviation of positive milk samples (>LOQ).

The AFM1 was analyzed in a total of 16,934 analytical samples of milk during the seven-year period from 2014 to 2020, and the mean values of the AFM1 concentrations in the cow and ewe’s milk collected during this period are reported in Figure 1. The level of AFM1 shows a decreasing trend after 2016, which was considered a year of crisis. Overall, the occurrence of AFM1 in milk showed an average percentage of left-censored data within milk samples of 44% (data not shown).

Concerning cow’s milk destined for use in drinking milk production (Table 1), the total number of samples analyzed was 3151, with a maximum of 551 in 2014 and a minimum of 377 in 2016. On average, 71.3% of the total samples were positive (>LOQ), with a maximum of 89.3% in 2016, while non-compliant samples (AFM1 ≥ 0.050 µg/kg) registered an average value of 0.86% and reached the highest percentage of 1.5% in 2015.

In same year, the highest concentration value was recorded, 0.146 µg/kg. LB values ranged from 0.006 (2018 and 2019) to 0.013 (2016); UB values ranged from 0.007 (2018) to 0.014 (2016). Table 2 shows the occurrence data of AFM1 in cow’s milk destined for cheese production. In the targeted period, 8529 samples were analyzed, ranging from 1492 in 2019 to 918 in 2016. Positive samples made up 68.2% of the total, with the highest value registered in 2018 (87.7%); incompliant samples made up 2.23%, ranging from 0.18% in 2018 to 0.47% in 2014. The highest concentration was reached in 2019, with a value of 0.208 µg/kg. LB and UB values ranged from 0.006 (2019 and 2020) to 0.010 (2016 and 2018) µg/kg and from 0.007 (2019) to 0.012 (2018) µg/kg, respectively.

The data concerning samples of ewe’s milk destined for use in cheesemaking are summarized in Table 3.

The total number of samples analyzed was 5254, varying from 608 in 2016 to 913 in 2019. The average percentage of positive samples was 27.1% (ranging from 9.90% to 49.0% in 2020 and 2016, respectively). The incompliance rate reached its maximum in 2016, with a value of 1.15%. The maximal concentration was registered in 2016, with a value of 0.239 µg/kg. The substitution method applied for left-censored data revealed the highest LB and UB values of 0.006 and 0.009 µg/kg, respectively.

### 3.1. Exposure Assessment

The potential exposure of the central Italian population to AFM1 through milk and dairy products was estimated using the 50th and 99th percentile consumption values of five individual age groups: toddlers, children, adolescents, adults, and the elderly. According to the data extrapolated from the Comprehensive Food Consumption Database of the EFSA, the average consumption of milk was 43.6 and 183.3, 10.9 and 37.2, 4.4 and 11.9, 2.9 and 8.5, and 3.0 and 8.1 g/kg bw/day for the 50th and 99th percentiles in each age class, respectively. Concerning cheese made from cow’s milk, the values of consumption elaborated upon in the present study were 1.04 and 1.53, 0.86 and 1.26, 0.55 and 0.70, 0.39 and 0.61, 0.35 and 0.62 g/kg bw/day for soft cheeses for toddlers, children, adolescents, adults, and the elderly for the 50th and 99th percentiles, respectively. For cheeses classified as semi-soft, the consumption data registered were 0.04 and 0.26, 0.04 and 0.21, 0.02 and 0.12, 0.02 and 0.10, and 0.01 and 0.10 g/kg bw/day for toddlers, children, adolescents, adults, and the elderly for the 50th and 99th percentiles of consumption, respectively. Regarding semi-hard cheeses, the values were 0.13 and 0.39, 0.11 and 0.32, 0.07 and 0.18, 0.05 and 0.15, and 0.04 and 0.16 g/kg bw/day for toddlers, children, adolescents, adults, and the elderly for the 50th and 99th percentiles of consumption, respectively. Ewe cheese consumption among the targeted population was 0.07 and 0.18, 0.06 and 0.21, 0.03 and 0.5, 0.03 and 0.5, 0.02 and 0.18 g/kg bw/day for toddlers, children, adolescents, adults, and the elderly for the 50th and 99th percentiles of consumption, respectively (Table S1). The values of dietary exposure (DE) to AFM1 of the selected consumer population, defined through the deterministic approach, are shown in Table 4.

In the seven-year period considered, the mean DE for average consumers was 0.00049 µg/kg bw/day for toddlers, 0.00015 µg/kg bw/day for children, 0.00007 µg/kg bw/day for adolescents, 0.00005 µg/kg bw/day for adults, and 0.00005 µg/kg bw/day for the elderly. Concerning the 99th percentile of consumption, the average DE was 0.00195 µg/kg bw/day for toddlers, 0.00045 µg/kg bw/day for children, 0.00017 µg/kg bw/day for adolescents, 0.00013 µg/kg bw/day for adults, and 0.00012 µg/kg bw/day for the elderly.

The contribution of each dairy category to AFM1 exposure for all age classes of the population is shown in Table 5. The main contributor in all classes is drinking milk, with the maximum value registered for high-consuming toddlers (95.22%), followed by soft cheeses made from cow’s milk, which reached the maximum value among average adults (31.48%). Ewe cheese contributed mainly in the high-consuming adult group.

### 3.2. Risk Characterization

The risk characterization is the final step of a risk assessment and implies the qualitative and/or quantitative estimation of the probability of occurrence and severity of known or potential adverse health effects in a given population on the basis of hazard identification, characterization, and exposure assessments. In the present study, the MOE values were calculated for each year and population class considered, for both mean and high consumers, as shown in Table 6. The average MOE values defined in the present study were 8498 and 2163 for toddlers, 27,301 and 9294 for children, 58,083 and 23,739 for adolescents, 85,277 and 32,437 for adults, and 87,572 and 34,900 for the elderly, all for the 50th and 99th percentiles of consumption, respectively. For all age classes, the lowest MOEs were registered in 2016 (Table 6).

### 3.3. Uncertainty Analysis

The more relevant sources of uncertainty affecting the dietary exposure assessment in the present study could mainly be attributed to the handling of non-detect, to the use of 99P of food consumption, and to the exclusion of some cheese categories with potential high levels of contamination. The qualitative analysis of the direction and magnitude of uncertainties revealed that the combined effect of the identified uncertainties might lead to an overall moderate overestimation of mean AFM1 exposure among the targeted population (Table S2).

## 4. Discussion

The concentration of AFM1 in the present work varied, ranging from <LOQ to values up to 50 ng/kg, corresponding to the limit established by EU legislation. These data are in agreement with data reported by other authors [34,35]. In this study, the percentages of non-compliant raw cow’s milk samples were slightly higher than the percentages reported in another study conducted in Italy (0.20%) by Serraino et al. [18] and in Spain by Cano-Sancho (0%) [36], but lower than those reported by other studies, such as in Greece (3.6%) [37] and Serbia (30%) in samples above the EU maximal residual limit [3]. In terms of the AFM1 levels in ewe’s milk, the percentage of samples below the EU limit is similar to that reported in literature [38], but remarkably lower than the percentages reported by Roussi [37] (6.7%).

Regardless of the species considered, incompliant milk batches were not used for human consumption. As reported above, the average AFM1 concentration during the seven-year period considered ranged from 0.009 to 0.015 µg/kg for raw cow’s milk and from 0.009 to 0.013 µg/kg for raw ewe’s milk (Figure 1). These values are in accordance with those reported in similar studies across Europe. For instance, a study in France reported an average value of 14.3 ng kg^−1^ in raw milk [39]. In Portugal, a mean value of 23.4 ng L^−1^ was reported in pasteurized milk, [34] and in Spain, a value of 9.69 ng L^−1^ was reported in UHT milk [40], with a range of 0.3–97.1 ng/kg for ewe’s milk [38]. The mean AFM1 concentration in the present study was lower in comparison to that found by Milićević et al. [3] in Serbia (5 to 1260 ng/kg; mean 71 ± 130). In some extra-EU countries, a higher incidence and higher level of noncompliance for AFM1 in milk and dairy products have been registered; however, it is important to consider that a higher maximal residual limit may be in force [41]. Some authors noted that the AFM1 contamination of raw milk is influenced by the season: in general, winter milk samples are found to be contaminated by higher AFM1 concentrations than summer samples. This seasonal variation may be attributable to the reduced availability of fresh green feed in colder periods; therefore, milk producers must increase their use of stored concentrated feedstuffs [42]. These feedstuffs are usually composed of corn, wheat, and cotton seeds that, if stored under inadequate conditions, may favor the development of toxigenic fungi such as those that belong to the *Aspergillus* genus. However, although seasonal variations have not been highlighted in the present study, in accordance with what reported by Bilandzic et al. [43], with the results showing that ewe’s milk was less contaminated than cow’s milk. This fact may not only relate to the peculiarities of the dairy species (different carryover rates), but also to the feed administered and the length of fodder storage. Sheep are generally fed fresh fodder, and, as previously reported, this feed usually has negligible levels of fungal contamination. On the other hand, cows are generally fed concentrates and stored fodder that are more subject to AFB1 contamination [43]. Concerning consumers’ exposure to AFM1, the DE results indicate that toddlers and children were the two most exposed groups of the population to AFM1, mainly due to their high milk intake and body weight ratio and to their relatively higher consumption of milk and dairy products. Moreover, as reported in Table 4 and the Results section, the DEs of the other three population groups (adolescents, adults, and the elderly) were remarkably lower. A comparison of the reported data with those in the literature must take into account the fact that few previous studies have considered both milk and dairy products in exposure assessments; however, the results of this study are in accordance with those of other authors [3,18,36,40]. In the context of risk characterization, the exposure values related to toddlers resulted in average MOE values below the safe limit of 10,000, both for mean and high consumers; however, for 50th percentile consumers, their MOE values in 2018, 2019, and 2020 were slightly above the limit of concern. As reported in Table 6, for children, the MOE was below the safe limit only from 2014 to 2017 for high consumers, resulting in a concerning average MOE. Although the MOE values referred to average consuming children are below 10,000, the risk characterization revealed that the exposure of toddlers and children to AFM1 raises health concerns. The exposure assessment of adolescents, adults and the elderly resulted in significantly higher MOE values, therefore attesting to the absence of health concerns in relation to these age classes [4]. These findings are in line with the results of the EFSA’s “Risk assessment of aflatoxins in food”, addressing the greater level of concern in relation to younger age groups, despite the fact that the study was performed on the basis of total diet and therefore the general exposure of the population was higher [1]. It is important to highlight that data on consumers’ AFM1 intake (all age groups in the 50th and 99th percentile) reflect a higher level of exposure during 2016 as a consequence of the higher AFM1 occurrence in milk registered in the same year [18]. As reported elsewhere, in 2016, the risk of mycotoxins raised and brought back public attention to the relevance of this food safety issue, albeit with lower levels of concern compared to previous crises, such as those in 2003 and 2013 [44,45]. It is likely that the higher levels of AFM1 contamination were a consequence of the particularly unusual climatic conditions (high temperatures and drought) [46] that characterized the summer of 2015. The combination of a more favorable climate and the implementation of some corrective measures at dairy farms may have led to a lower occurrence of AFM1 contamination in subsequent years.

## 5. Conclusions

Our risk assessment of AFM1 dietary exposure from cow and ewe’s milk and milk-based products in the central Italian population evokes a high level of concern from a public health point of view with regard to the youngest classes of consumers. This outcome should reinforce the consciousness of food business operators and risk management authorities in terms of the global health implications of this food safety issue. Even though a low level of concern has emerged for the other age groups (adolescents, adults, and the elderly) and deterministic exposure assessments are characterized by an inherently conservative nature, this should not preclude the application of risk management measures to reduce exposure at all ages. AFM1 represents an unavoidable contaminant, but the common scientific consensus is that there may not be a threshold dose and that some degree of risk may exist at any level of exposure. Moreover, in light of the high variability of the factors influencing the aflatoxin contamination of foodstuffs, and in light of the irreversible climate change we are experiencing, there is a strong need for the constant monitoring of the occurrence of AFM1 in milk by inspection authorities, as well as for the regular updating of exposure assessments.

## Figures and Tables

**Table 1:** Incidence of positive and noncompliant (n.c.) samples, AFM1 concentration range (µg/kg), and average lower bound LB and upper bound (UB) values in samples of cow’s milk destined for use in drinking milk collected within the Italian National Residue Control Plan from 2014 to 2020.

	No.	% Positive	% n.c. *	Min	Max	LB	UB
2014	551	66.4	0.9	<0.005	0.073	0.009	0.011
2015	478	73.2	1.5	<0.005	0.146	0.011	0.013
2016	377	89.3	0.8	<0.005	0.068	0.013	0.014
2017	442	80.0	-	<0.005	0.041	0.009	0.010
2018	402	69.2	-	<0.005	0.028	0.006	0.007
2019	437	56.9	0.68	<0.005	0.069	0.006	0.008
2020	464	64.3	0.43	<0.005	0.097	0.007	0.008
Average	450	71.3	0.86	<0.005	0.075	0.009	0.010

* n.c. = non-compliant.

**Table 2:** Incidence of positive and noncompliant (n.c.) samples, AFM1 concentration range (µg/kg), and average lower bound (LB) and upper bound (UB) values in samples of cow’s milk destined for use in cheesemaking collected within the Italian National Residue Control Plan from 2014 to 2020.

	No.	% Positive	% n.c.*	Min	Max	LB	UB
2014	1289	59.9	0.47	<0.005	0.139	0.007	0.010
2015	1134	66.6	0.44	<0.005	0.095	0.009	0.011
2016	918	80.3	0.32	<0.005	0.090	0.010	0.011
2017	1189	76.3	0.34	<0.005	0.091	0.008	0.009
2018	1121	87.7	0.18	<0.005	0.192	0.010	0.012
2019	1492	46.8	0.26	<0.005	0.208	0.006	0.007
2020	1386	59.7	0.22	<0.005	0.127	0.006	0.008
Average	1218	68.2	0.32	<0.005	0.135	0.008	0.010

* n.c. = non-compliant.

**Table 3:** Incidence of positive and noncompliant (n.c.) samples, AFM1 concentration range (µg/kg), and average lower bound (LB) and upper bound (UB) values in samples of ewe’s milk destined for use in cheesemaking collected within the Italian National Residue Control Plan from 2014 to 2020.

	No.	% Positive	% n.c.*	Min	Max	LB	UB
2014	679	34.6	0.74	<0.005	0.112	0.004	0.007
2015	666	19.8	0.30	<0.005	0.139	0.002	0.006
2016	608	49.0	1.15	<0.005	0.239	0.006	0.009
2017	763	33.42	0.66	<0.005	0.084	0.003	0.007
2018	777	24.10	-	<0.005	0.037	0.002	0.006
2019	913	18.84	0.33	<0.005	0.096	0.002	0.006
2020	848	9.90	0.24	<0.005	0.084	0.001	0.006
Average	751	27.1	0.57	<0.005	0.113	0.003	0.006

* n.c. = non-compliant.

**Table 4:** Values of average dietary exposure to AFM1 (g/kg bw/day) for the five age classes considered and for mean (50th percentile, 50P) and high consumers (99th percentile, -99P).

	**Dietary Exposure 50P**				
	**Toddlers**	**Children**	**Adolescents**	**Adults**	**Elderly**
2014	0.00053	0.00016	0.00008	0.00005	0.00005
2015	0.00063	0.00019	0.00009	0.00006	0.00006
2016	0.00067	0.00020	0.00009	0.00006	0.00006
2017	0.00049	0.00015	0.00007	0.00005	0.00005
2018	0.00037	0.00013	0.00006	0.00004	0.00004
2019	0.00039	0.00012	0.00005	0.00004	0.00004
2020	0.00039	0.00012	0.00006	0.00004	0.00004
Average	0.00049	0.00015	0.00007	0.00005	0.00005
	**Dietary Exposure 99P**				
	**Toddlers**	**Children**	**Adolescents**	**Adults**	**Elderly**
2014	0.00211	0.00048	0.00019	0.00014	0.00013
2015	0.00249	0.00057	0.00021	0.00015	0.00015
2016	0.00267	0.00060	0.00023	0.00017	0.00016
2017	0.00192	0.00044	0.00017	0.00013	0.00012
2018	0.00140	0.00035	0.00015	0.00011	0.00010
2019	0.00153	0.00035	0.00014	0.00010	0.00009
2020	0.00154	0.00036	0.00014	0.00010	0.00010
Average	0.00195	0.00045	0.00017	0.00013	0.00012

**Table 5:** Contribution (%) of each dairy category to the exposure of different classes of the population and for mean (50th percentile, 50P) and high consumers (99th percentile, -99P).

	Toddlers		Children		Adolescents		Adults		Elderly	
	50P	99P	50P	99P	50P	99P	50P	99P	50P	99P
Cow’s drinking milk	89.38	95.22	72.59	82.71	62.61	69.03	60.63	63.68	64.32	66.79
Soft cow cheese	8.15	3.04	21.92	10.76	29.89	15.45	31.48	17.48	28.53	19.53
Semi-soft cow cheese	0.78	0.52	0.93	1.82	1.26	2.62	1.33	2.96	1.21	3.31
Hard/semi-hard cow cheese	1.29	0.97	3.48	3.42	4.74	4.91	4.99	5.56	4.53	6.21
Ewe cheese	0.41	0.26	1.09	1.29	1.49	7.99	1.57	10.32	1.42	4.16

**Table 6:** Estimation of the margins of exposure (MOEs) for mean (50P) and high (99P) percentiles. of population exposure to AFM1. The calculated MOE values were below 10,000, which raises a health concern, shown in red.

	**MOE 50P**				
	**Toddlers**	**Children**	**Adolescents**	**Adults**	**Elderly**
2014	7526	24,555	52,738	77,565	79,392
2015	6377	21,211	45,912	67,631	69,024
2016	5954	19,978	43,480	64,119	65,305
2017	8229	27,050	58,129	85,503	87,500
2018	10,782	31,420	63,135	91,675	96,109
2019	10,377	34,022	73,279	107,836	110,260
2020	10,244	32,868	69,905	102,614	105,412
Average	8498	27,301	58,083	85,277	87,572
	**MOE 99P**				
	**Toddlers**	**Children**	**Adolescents**	**Adults**	**Elderly**
2014	1894	8258	21,412	29,293	31,490
2015	1608	7064	18,808	25,920	27,450
2016	1496	6628	17,330	23,660	25,623
2017	2083	9098	23,422	31,948	34,583
2018	2864	11,409	27,509	37,675	39,850
2019	2607	11,422	29,276	39,831	43,391
2020	2591	11,182	28,416	38,732	41,910
Average	2163	9294	23,739	32,437	34,900

## Data Availability

The datasets generated for this study are available on request to the corresponding author.

## Supplementary Materials

The following are available online at https://www.mdpi.com/article/10.3390/foods10071529/s1, Table S1. Detailed consumption data of dairy products of the entire central Italy population (g/kg bw/day). Table S2. The direction and magnitude of individual uncertainty and the combined effect of all the uncertainties affecting the exposure assessment.
