# Supplementary material for: A Study of the Occurrence of Aflatoxin M1 in Milk Supply Chain over a Seven-Year Period (2014–2020): Human Exposure Assessment and Risk Characterization in the Population of Central Italy
Source: Foods. 2021 Jul 2;10(7):1529. doi: 10.3390/foods10071529 (PMC8304948; doi:10.3390/foods10071529)
Supplement: Supplementary file 1 [file foods-10-01529-s001.zip › foods-1267496supplement.pdf]

Table S1. Detailed consumption data of dairy products of entire central Italy population (g /kg bw/ day)

	Toddler		Children		Adolescents		Adults		Elderly	
	50P	99P	50P	99P	50P	99P	50P	99P	50P	99P
Milk	43.60	183.30	10.90	37.20	4.40	11.90	2.90	8.50	3.00	8.10
Soft cheese	1.04	1.53	0.86	1.26	0.55	0.70	0.39	0.61	0.35	0.62
Semi-soft cheese	0.04	0.26	0.04	0.21	0.02	0.12	0.02	0.10	0.01	0.10
Semi-hard cheese	0.13	0.39	0.11	0.32	0.07	0.18	0.05	0.15	0.04	0.16
Ewe cheese	0.07	0.18	0.06	0.21	0.03	0.50	0.03	0.50	0.02	0.18

Table S2. The direction and magnitude of individual uncertainty and the combined effect of all the uncertainties affecting the exposure assessment

**Source of uncertainty**	**Direction and magnitude**
Measurement uncertainty (analytical variation, and measurements below the limit of detection or quantification)	+/-
Size and variability of analytical data set	+/-
Influence of non-detects in analysis	++/--
Treatment of non-detects for exposure assessment	+
Default bodyweight for age groups with no gender diversification	++/--
Consumer population selection bias	from +/- to +++/---
Extrapolation of cheese contamination (use of concentration factors)	from +/− to ++/−−
Conservative assumption that individual consumers consume food categories at 99th percentile	++
Exclusion of some cheese categories with high levels of contamination	-
**Qualitative evaluation of overall effect of identified uncertainties:**	++
